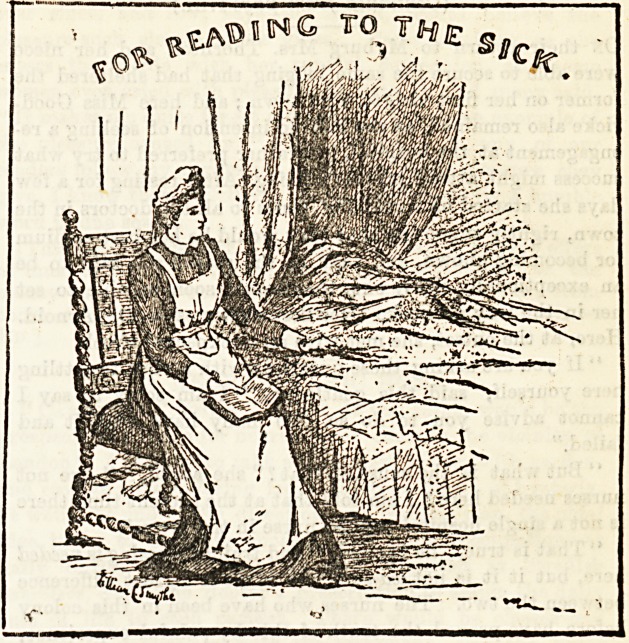# The Hospital Nursing Supplement

**Published:** 1891-12-12

**Authors:** 


### "The Hospital" Nursing Mirror.

Being the Extra Nursing Supplement of "The Hospital" Newspaper.

Contributions for this Supplement should be addressed to the Editor, The Hospital, 140, Strand, London, W.C., and should have the word "Nursing" plainly written in left-hand top corner of the envelope.

### En Passant.

LEPER CHILDREN.—It will interest those of our readers who responded to Miss Gethen's appeal in the "Mirror" of July 25th, to know that early in November a famous box was sent to Colombo, and we hope its contents will be distributed to the poor children at the Leper Hospital at Christmas. There were a famous lot of scrap-books and large pictures, and a few boxes of toys and dolls from various kind friends. Amongst those who contributed offerings were: Mrs. Stafford Smith, Miss K. Black, Mrs. McKenna, Mrs. Goldingham, Miss Newsham, Miss Fanny Lamport, Miss Abdy, Miss Appleby, Miss M. F. Smith, Miss Edwards, a Friend at Hunstanton, and others. Early in the New Year we hope to have a letter from the chaplain, acknowledging the safe arrival of our box.

SHORT ITEMS.—Miss Nightingale has written a preface to the life of Behramji M. Malabari.—Professor Jowett is said to owe his recovery largely to the clever and sympathetic nursing of Mrs. Green, widow of the historian, who, since her husband's death, has devoted much of her time to nursing.—The Nurses' Hostel, which was opened by Miss Wood two years ago, is so successful it is to be enlarged. A nurse writes to tell us from her personal experience how comfortable the hostel is.—We shall be glad to hear at once from Matrons or Sisters of metropolitan hospitals who are in need of presents for their adult patients on Christmas Day.—Lady Harris has become patroness of the Mofussil Nursing Association, which is being formed in India, to provide private nurses for sick Europeans.—Letters have reached us from Ventnor and elsewhere pointing out that "The Daily Nurse" has long existed in certain towns, in spite of the fact that she is announced as just "created" by Miss Wood. An amusing caricature also reaches us, but is too impolite to publish.

BELFAST NURSES' HOME.—This excellent institution held its annual meeting on November 27th, the Countess of Shaftesbury in the chair. The Home is a comfortable one, and the nurses receive lectures, and newspapers are supplied. Finances are low, but we agree with Lady Shaftesbury that the charges for the nurses' services is too low; we quote from the Countess's speech: "On looking over the moneys paid for private nursing in this country compared with what was paid in England, I was wondering whether it would be possible to increase the prices of private nursing per week, and to have them on a running scale, say from a guinea to thirty shillings. An increase of 2s. per week would not be felt by patients, and, considering the large number of persons who require private nurses, the result would be a great improvement in the financial condition of the Home. This strikes me as an easy way of getting rid of your financial liabilities, which must increase year by year. At all events, you know more about this matter than I do, and I am sure some plan will be devised whereby the Home will be placed on a firm financial basis. I have great pleasure in moving the adoption of this report. I hope the Home will flourish more in the future than it has in the past, and that every difficulty may be removed from its path. I trust that its efforts on behalf of the poor, which is a new departure in connexion with the institution, will be increased. I think it rather a good thing, if you will allow me to say so, that ladies from England do not come over here. I think we should train Irishwomen to do Irish work."

LINCOLN INSTITUTION.—A meeting has been held at Miss Bromhead's to consider how to raise funds for this excellent institution, and the Bishop proposed the following resolution: "That this meeting approves the proposal to hold a bazaar in November, 1892, in aid of the funds of the Nursing Institution, and pledges itself to use its utmost endeavours to make the same a success." He knew that sometimes people urged objections to bazaars just as they did to dancing. He knew that evil might be made out of anything, even out of bazaars, but he had been to many bazaars, but he never got any evil from them. On the contrary he had seen a great deal of good come out of bazaars, for they gave to many people in different positions in life the opportunity of taking part in a great work. He ventured to hope that resolution would be carried, because he believed the object was of the highest Christian kind. Canon Harvey seconded the resolution. Speaking with regard to the work of the Nurses' Institution, he said that the nursing of the sick poor cost the institution a sum of no less than £319 a year; that sum was paid for the maintenance of seven district nurses. The resolution was carried, a committee was appointed, and a most successful meeting was closed with the usual votes of thanks.

SALVATION ARMY NURSES.—A correspondent has been kindly enquiring into the amount of training given to the slum sisters of the Salvation Army, who have now proclaimed their willingness to undertake nursing work. We quote from a letter from the Rescue Head Quarters: "So far as our own officers are concerned, those of them who are trained nurses come either from the Middlesex Hospital, Nottingham, St. George's, Tottenham Deaconess Institution, and some with two years' infirmary experience." This sounds satisfactory, and we sincerely hope the Salvation Army will allow none of its workers to adopt the title of nurse unless they have spent one year in a good hospital or infirmary. Let all the slum sisters minister so far as they can, and, according to their knowledge, to the sick who come under their care, but, in perfect fairness, let them not be called "nurse" unless they have had a year's training. It is too much the habit of religious societies in their eagerness for the welfare of the soul to leave the bodies of their converts to the care of half-taught women doctors and half-trained nurses. This brings discredit on them in the long run, and is bad policy, furnishing stones for the adversary, and giving its followers a bad standard in place of that thoroughness which ought to mark their every deed.

GLASGOW ASSOCIATION.—On November 20th was held the annual meeting of the Glasgow Sick Poor and Private Nursing Association, when a good report was submitted. The whole nursing staff of the Association at the close of the year (October 31st), exclusive of the Superintendents at the home and the districts, numbered 66. The work among the poor still continued to be carried on by the district nurses of the Association. During the past year these ministrations were still further extended by the addition of another district (Maryhill), so that there were now 15 trained nurses visiting the patients daily, with nine assistant nurses. During the year 63,220 visits were paid, compared with 62,113 in the previous year. In some cases modified fees were paid. The private nursing staff of the Association had been well employed during the year. There were at present 43 nurses on this branch of the Association, and the number of cases on the books last year was 477, as against 415 in the previous year. The ordinary expenditure for the year was £3,467, and the ordinary receipts £3,230. A thousand pounds is still needed to complete the building as a memorial to the late Mrs. Higginbotham. The Lady Superintendent and the nurses are to be congratulated on their year's work.

### The Pen-Bron Hospital.

*Translated by permission from the French of M. Pierre Loti.*

(*Concluded from page* lvi.)

What kind of faces are these scrofulous children likely to have?

Why! Faces like everybody else; sometimes even, to my great surprise, very pretty faces; full and round, which imitate health. How brown and burnt by the sun they are! On their cheeks, like real little fisher children, they have the patina of the sea. It seems as if they had borrowed that goodly tan of the wind and the sun, which makes them look so robust, from sailors' children. What a complete surprise to find them thus! But on a nearer view there are certain details which make one shudder; under the wide little peasant trousers are legs cruelly twisted and distorted, bowed tibias. Under the little jackets are stiff stays to support a softened spine, which but for them would give way. Then there are still great wounds, barely healed, in the flesh, hollow and dreadful scars; in short, a whole series of mysterious and gruesome phenomena.

In spite of all there is joy and laughter in most eyes. One feels that confidence and hope have reasserted themselves among these little, wasted invalids, now conscious of an unlooked for renewal of life in their weakened bodies.

M. Pallu, who is with me, calls them up one by one, for he is quite proud to show me such well-bronzed cheeks. Poor children! They show me their scars without any feeling of shame, and I hear from each the sad story of his past sufferings. Here is one who had an open wound in his side under the arm for six years. The cavity was growing larger, and hospital treatment was of no avail. He has spent four or five months at Pen-Bron, and the trouble is over—the wound has closed. He draws aside his little shirt to show me the place, where there is now nothing to be seen but a long scar, still rather red.

Another little fellow, of about ten, had lain for four years on a hospital bed, stretched out in a sort of box, with Pott's Disease, a disease of which I had never even heard; yet its very name, I know not why, has an unpleasant sound and chills my blood. It affects the spinal column, the bones of which are no longer united. The c artilage between them is wasted away, and the little frame of the child, if left to itself, would collapse like a Venetian lamp taken off its hook. The child who had that illness stands erect before me, and two or three days ago the stiff stays which supported his back during his first walks were removed. He no longer needs them, and will scarcely be at all deformed.

All the children have such things to show or tell me with a cheerful naiveté and an air of absolute confidence in their speedy and effectual recovery. The fine salt air of Pen-Bron absorbs all such insidious human decomposition, almost as surely as the warm winds of summer dry up putrid swamps or the leakage and moisture of walls.

We then entered the hospital, which, during the day, is almost empty. It is a very old building, transformed by M. Pallu out of an old salt store. In carrying out his plan, he has needed the greatest determination and persistence. It is true that the larger part of the expenses have been met by donations. But a hundred thousand francs or thereabouts are not collected for a work having in it so little to attract, at first sight, without much trouble, and many a rebuff.

The Peu-Bron Hospital has at the present time about one hundred beds, one hundred children's beds, some of them not much bigger than a cradle. The white-washed wards open on either side to the sea; through the windows, as in a floating-house, one sees only vast ocean spaces, vast, ever-changing horizons, with fishing craft sailing by. Then the chapel, with its oaken vaulting and extreme simplicity, is like a ship's chapel.

Those little patients who have not long arrived, and are not yet able to go out, instead of looking at bare grey walls, as in most ordinary hospitals, are amused, as they lie, with watching the passing boats, and breathe, even in their cots, the vitalising air of the open sea. Compared with the older inmates, these new comers are pallid, have a waxy transparence of complexion, and eyes that are too large and hollow. But their tme of probatiion in the wards is not usually very long. As soon as possible, *coûte que coûte*, they are sent out into the sun to inhale the salt smell of the waters.

A special kind of boat, even, in which they are laid, has been constructed, a kind of floating bed, to take them on to the lagoon. Through an open window I am shown the strange, feeble, little squadron which puts off from the shore towed by a boat. In three of these raft-beds are pale-faced children, while the Chaplain, seated in the boat with a book to beguile the long hours of daily anchorage, is of the company.

Among those children who cannot go out yet there are some very wizened and pallid faces, certainly, which make me sadder than the faces of dead children. But I am greeted by all with a smile, which surely has been enjoined on them; before I came they must have been told that I was a person devoted to their interests. Perhaps in their dreamy imagination they endow me with some half-magical, beneficent powers. I know that their kind glances seem to bind me over still more to do my best for the hospital. Here and there toys are lying upon the beds—very simple ones; dolls for the little girls, or rather legless trunks clothed in cotton dressing-gowns. In another place a little boy of four or five, his legs in splints, and with weights fastened to his feet to prevent his softened bones from contracting, is setting up some cardboard soldiers, given him by the good Sister, in rows on the sheet. Then my glance is rivetted by a charming little white-and-pink maiden of some twelve summers, with singularly refined features, who does not play at anything, but her head upon the spotlessly-white pillow seems plunged in some melancholy dream.

I ask, What is this pretty little creature's illness? They tell me it is that horrible Pott's Disease in its last stage, and that they fear it is very late to hope for a cure. I am strangely moved by the expression of her face. It is like an appeal, a sorrowful entreaty, like a cry of prophetic and fathomless despair. Moreover, those agonised prayers dumbly and briefly present in the eyes of sick children and of the aged poor, or even in the eyes of trembling animals, beaten and suffering, the dispossessed of the earth, affect me more, I confess, than prayers or tears. Ah! that poor little girl! And I had said once, in speaking of the Pen-Bron children, "Better leave them to die"!

It is easy to make such general and vague statements before seeing for oneself, but when it comes to its individual application the thought at once seems monstrous, impossible. Then by what right, when the means of prevention are here, do we let intelligent, clear young eyes like those depart for the mysterious unknown, eyes which have scarcely opened upon life?

Well if the development of these hospitals into a great work of national regeneration be an impossible dream! Still, for the sake of restoring to health some such small creatures as those I have seen, it would be worth the trouble a hundred times over to continue and extend the scheme. But, with money—a great deal of money—of course, the dream itself might be realised. Behind the existing hospital stretches that interminable sandy peninsula like a yellowish riband, farther than the eye can reach, between the blue waters of the sea, and the yet bluer waters of the salt lagoon. It is on this incomparable site that M. Pallu, the founder of Pen-Bron, dreams of extending his rows of white beds in a building kilometres long, where thousands of enervated children may come to have, like sailors, wide chests and strong muscles.

. . . . . . . . . .

Above all, let it not be thought that I have lent my pen unawares to some self-interested speculation. There must be no mistake as to that point. The founder of Pen-Bron gave his money as well as his time and energy. There is an Administrative Committee which is not remunerated; a Council composed of people who, when a deficit occurs in the funds, meet it out of their private purse; doctors who are not paid, and who come every day from Nantes out of pure devotion to the cause; Sisters of Charity who are truly admirable. One incident will serve to characterise the Sister Superior without further description. Owing to lack of funds, the soiled bandages used for the patients cannot be burnt; they have to be washed and used again. The hired women refused this disgusting task; so said the Sister, simply, "I will wash them." Now, she washes them daily in her hour for rest.

Thus a band of warm-hearted people have come together, are united by a common faith in the work they have begun and upheld amid terrible difficulty by the marvellous results attained. They have set their hopes on me—on my being able to say something to make their work known, and I tremble lest they should be disappointed because it is not, I am well aware, attractive at first sight.

They need money, not only to carry out the great idea they dream of, a wholesale regeneration for the children of France, but even to meet the most urgent present distress. Every day for want of space they are forced to turn away parents who have come to entreat admission for their children. Would that my words might be heard and bring some gifts! Or, if at least I could inspire those whom I may have failed to convince with the curiosity during their seaside holiday to visit Pen-Bron. I feel sure that after seeing it they would be won over as I was, and that they would give.

### The Princess of Wales and the Nurses.

SUBSCRIPTIONS of 2s. 6d. and 2s. respectively are acknowledged from Sister Tulloh and Nurse E. Bishop.

In this week's issue of *The Gentlewoman* (published Thursday, December 10), will be given a large reproduction of "The Screen of Photographs of the Nurses of the Royal National Pension Fund," presented to the Princess of Wales, which has been reproduced expressly for *The Gentlewoman* by the gracious permission of Her Royal Highness. Copies may be obtained at every bookstall and newsagent in the kingdom. Price 6d.

### H.R.H. The Princess of Wales.

WE publish this week, as a special supplement to the "Nursing Mirror," an autograph letter from H.R.H. the Princess of Wales, expressing Her Royal Highness's "warmest thanks for the beautiful screen given her as a birthday present by so many nurses of the Royal National Pension Fund, and to assure them that she will always value most highly this token of their kind feeling towards one who takes the deepest interest in their work and welfare." A fac-simile of the letter is issued with each number of THE HOSPITAL. Every nurse may thus become possessed of a copy of the gracious letter of the Royal President of their pension fund. We are quite sure that the letter will be highly valued by the nurses, and will be preserved as a lasting memento of kindness truly royal, and of gratitude on the nurses' part as spontaneous as it is profound.

### THE GOOD FIGHT.

LIFE is often called a battlefield, for in it we have to fight, as there is constant war being waged between good and evil. Now we must be on one side or the other, and the question is, Which have we already chosen? Have we taken up arms under the banner of the Great King, or have we enrolled ourselves among the followers of the prince of darkness? Such thoughts should come into our minds as we lie on a sick bed, for the query is a very important one, and we have time and quiet in which to find out the answer. If we are not with Christ we are against Him; if we do not oppose with all our hearts wickedness and sloth and evil speaking, lying, and slandering, and worse sins still, all of which He hates, we are passively consenting to give the enemy an advantage even though we do not commit the sins ourselves. Let us see what a good soldier should be like and how near we come up to the standard.

A good soldier is brave, never ashamed of the flag under which he is fighting; he stands up for its glory and renown before all the world. Are we bold when we are scoffed at, or do we fear and dissemble when men call us pious or religious? Can we bear pain and afflictions patiently because the Great Captain of our salvation was made "perfect through suffering"? Insfine, can we answer "Yes" to this question?

Again, a good soldier is obedient to orders, though he does not know why or wherefore they are given; he believes his commander, and acts accordingly. We should take our Father's orders in the same spirit, whether He sends us to work in His vineyard, or bids us wait patiently till He has prepared us by tribulation for His army in heaven. Then a soldier should be on the alert, lest he be surprised by the enemy, and Christ tells us we are to watch and pray lest we enter into temptation. A soldier is bound to keep his weapons clean and ready for use, and to be prompt to use them. We also should be armed with the breastplate of righteousness and the helmet of salvation and the shield of faith, and these must be kept bright and pure, so that we may be safe from the assaults of temptation, and with the sword of the Spirit, which is the word of God, we shall lay low our foes.

Let us choose Him, then, for our leader, "Whose banner over us is Love"; let us so fight the good fight here, that hereafter may be laid up for us a crown of righteousness.

The Son of God goes forth to war,
A kingly crown to gain,
His blood-red banner streams afar;
Who follows in His train?

Who best can drink the cup of woe,
Triumphant over pain,
Who patient bears his cross below,
*He* follows in His train.

### A Nurse in Natal.

(*Continued from page* lviii.)

On their return to Myburg Mrs. Thornton and her niece were able to secure the same lodging that had sheltered the former on her first visit to that town; and here Miss Goodricke also remained, as she had no intention of seeking a re-engagement at the hospital, but rather preferred to try what success might attend private nursing. After resting for a few days she started on a round of visits to all the doctors in the town, rightly supposing that they would be her best medium for becoming known, and that, as the season was said to be an exceptionally sickly one, they would soon be able to set her in the way of work. Her first visit was to Dr. Arnold. Here, at the outset, she met with an unexpected check.

"If you are asking these questions with a view to settling here yourself," said this gentleman, "I am sorry to say I cannot advise you to do so. So many have tried it and failed."

"But what is the cause of that?" she asked. "Are not nurses needed here? I am told that at the present time there is not a single hospital-trained nurse in the town."

"That is true," he replied, "and trained nursing is *needed* here, but it it is not *wanted*, and there is a great difference between the two. The nurses who have been in this colony before have proved the truth of this by painful experience, for they have not been able to obtain employment to support them. Of course, I do not know your private circumstances, but I am assuming that you wish to follow your profession here as a means of making a living, and, if so, I would strongly advise you to think twice about it. For if you only get occasional cases, and are then without work for weeks together, will it not be a very serious thing for you? How are you to live in the meantime? You must not think," he continued, "that I speak unfeelingly, or from want of interest in the subject. I think it is the truest kindness to speak plainly. The fact is, Miss Goodricke, people here do not like nurses—they prefer to be nursed by their own friends. And then, of course, there are some who *would* be glad to get a nurse in time of sickness, but they simply cannot afford it. They find it hard enough to pay the doctor, but they look upon him as indispensable. To pay a nurse as well would be, in many cases, impossible. However, if you elect to remain here, I will do my best for you by recommending you to my patients. But you must know yourself that, however glad a doctor might be to have a properly qualified nurse for his cases, he cannot compel his patient to see the matter in the same light. The homely proverb applies here—One may take a horse to the water, but cannot make him drink!"

After this conversation, Miss Goodricke proceeded, disappointed and perplexed, to the other medical men in the town. They all spoke in the same strain; all would be glad to avail themselves of her services, but all feared she would not find much opening in Natal. One doctor only thought he would be able to find her plenty of employment, but the result proved that this hopefulness was merely the effect of a more sanguine temperament. In contradistinction to the discouraging opinions of the doctors were the answers given by those few people whose acquaintance Miss Goodricke had made, and to whom she spoke of her plans. "Do well? Oh, yes, they were sure she would do well! Nurses were so badly needed. The poor Gibsons had been in such trouble; three of their children ill with diphtheria, and the mother quite knocked up. How nice for them to have had Miss Goodricke, though it was true Mrs. Gibson had the greatest prejudices against nurses." Or, "How thankful they themselves should have been for her when they were so ill ten years ago; and if they ever should be ill again they were sure they would only be too pleased to send for her. It would be such a comfort now to know that there really was a good nurse in the town in case one should ever need her. Had she heard of the Martins on the Vlei? Poor creatures! The father and mother both ill of fever, and three little children with no one to look after them. They were too poor even to pay a doctor, but what a boon it would have been to them to have had Miss Goodricke! Succeed? Of course she would, she was bound to succeed," &c., &c. These illogical speeches sounded very pleasant and hopeful, but when one came to look into them there was not much to take hold of. Most of the instances mentioned were hypothetical cases, and others were of people so indigent that, however they might appreciate a nurse, they had it not in their power to repay her services.

It was soon proved that the doctors really knew what they talked about, for during six months' residence in the town Miss Goodricke had only eight weeks' employment in several small cases, three of which were of a nature not requiring a trained nurse at all. The remaining sixteen weeks were wholly unoccupied. This paucity of cases was the more striking when it was taken into consideration that this was acknowledged by the doctors to be the most sickly season that many of them could remember.

(*To be continued.*)

### The Nurses' Conversazione.

There was a very large gathering at the Prince's Hall last week, for the fourth annual soirée of the Royal British Nurses' Association. There was, alas! no Corney Grain this year, but laughter gave way to loyalty, and the Princess Christian, as President of the Association, had a very hearty reception. We noticed both naval and military sisters in their pretty capes, we noticed many wearers of the Barts'. gold medal, many wearers of the pretty tulle ruches which form the caps of the St. John's workers, and at least two Sisters from the London Fever in the flat plain cap which we consider the most sensible and the most becoming ever invented. The Prince's Hall was, in fact, filled by an animated and merry crowd of nurses—they swarmed into the gallery, and they gathered on the platform; they were everywhere, and always pleasant to see and eager to be behold. Their Royal resident was very punctual, and her entrance was greeted by the singing of the National Anthem by Miss East, of the Queen's Square Hospital, who looked very handsome in a yellow satin gown. Gathered on the platform were well-known Matrons; Mrs. Bedford Fenwick was of course to the fore, and was wearing uniform; Miss C. J. Wood was not in uniform; Miss Mollett, looking none the worse for her trials at Johannesberg; Miss Ridley; Miss Stewart, of St. Bartholomew's; Miss Barton, of the Royal Free; and Miss Lancaster. Princess Christian was dressed in a rich crimson brocade trimmed with gold fringe, and was accompanied by Princess Victoria, Prince Christian, Lady Jeune, Miss Lock, and many others. Sir William Savory received Her Royal Highness, and asked her acceptance of the gold badge of the Association, which the Princess promptly pinned to her breast. The badge consists of a Maltese cross and circle; on the circle is the name of the Association; there is a clasp on which is the motto, "Steadfast and True." The members of the Council have the right to the badge in silver, the other members to the badge in bronze. About three hundred and fifty only came forward to receive the badge, and the fact that Miss Wood and other original supporters of the Association were not amongst them was much commented on. Gossip was also rife as to the scene at the Council meeting the week before the soirée, and there was a general feeling that things must shortly come to a crisis, and the management of the Association be reformed. The following doctors were well to the fore on Friday: Sir Dyce Duckworth, Sir Henry Thompson, Sir Joseph Fayrer, Mr. Gant (wearing his badge, and besieged by Royal Free Sisters), Dr. Cullingworth, Dr. Alhans, and Dr. Guthrie. The church was represented by the Rev. Somerville Lushington. After presenting the badges, Princess Christian walked round the hall and round the galleries, finally taking her departure about half-past ten. The refreshment department was not so well managed as usual, still only expressions of pleasure, and regret that the evening was over, could be heard from the departing nurses, who, donning cloaks and veils, went out into the night—and back to far other scenes than that in Prince's Hall.

### Everybody's Opinion.

[*Correspondence on all subjects is invited, but we cannot in any way be responsible for the opinions expressed by our correspondents. No communications can be entertained if the name and address of the correspondent is not given, or unless one side of the paper only be written on.*]

### HOSPITALS IN TOWNS.

"M. D." writes: In reference to the paragraph in last week's Hospital, respecting the increasing unsuitability of London as a place of residence for the sick, allow me to direct your attention to enclosed letter, which was published some few months since in the *Manchester Guardian.* All your remarks with reference to the impurity of the air, noise, and gloomy surroundings of most metropolitan hospitals are tenfold more emphatically applicable to such institutions in Manchester, and especially to the Royal Infirmary in that city. It seems almost incredible that several medical men, distinguished amongst their fellow-townsmen for professional skill and knowledge of theoretical sanitary science, should persist in endeavouring to persuade the Infirmary Board to extend that insanitary abode for the sick and suffering on its present site. And not only are some of the Royal Infirmary medical staff so obtuse, but the authorities of St. Mary's Hospital (Manchester) for Women are still more indifferent to the first necessities of women suffering acutely from diseases peculiar to themselves. There surely can be no plausible reason for building a women's hospital in one of the noisiest, dirtiest thoroughfares of a great city, except for the sole convenience of the medical staff connected with it, and yet a new site for this institution has been selected and—I believe—actually bought, quite close to a railway station, the much-abused Theatre of Varieties, and in a comparatively narrow thoroughfare crowded continually with passing tramcars and other heavy traffic; and, moreover, in a valley in which the almost perpetual fall of fog and smoke peculiar to Manchester hangs densely and most oppressively. This Institution is not a Maternity Hospital, and does not receive general surgical cases of accident; therefore, what excuse can there be for selecting such a position for it? The site of the present justly condemned hospital is bad enough, but the new one is worse. The authorities of the Southern Hospital—also for women—have, on the contrary, chosen a quiet, retired spot, most conveniently situated, and with plenty of open space around, for their new haven for their afflicted sisters. But I am told that certain wealthy residents who live in this desirable locality are very irate to find that their dignified seclusion is to be trespassed upon by such vulgar neighbours as the suffering poor and their attendants. One lady, indeed, was heard to say, *apropos* to this new departure, that she "would not like to live near a hospital because the nurses would be sure to gossip with her servants and unsettle them." Comment on this speech, so uncomplimentary to nurses generally and to Manchester nurses in particular, is unnecessary.

### A WELCOME GIFT FOR HOSPITALS.

"Amy" writes: Christmas is a time that appeals to hearts and purses, and whilst the former are warm with benevolent desires, let me suggest a welcome little gift for a hospital. The happy effect of music on many a weary patient is beyond dispute. The difficulty, however, of providing it conveniently and judiciously is very great. St. Cecilia choirs are not always desirabilities nor possibilities, but against the humble musical box, provided it be of fair quality, what can be said? I have a dear old musical box, just a little older than myself, which owing to my frequent absence from home, was somewhat wasting its sweetness on the desert air. A friend, a constant visitor to our provincial hospital, accepted with avidity my offer to lend her my musical box for a short time to amuse the patients. It has continued its mission ever since, and few, says my friend, would believe the pleasure such simple music gives. Faces brighten as she enters the wards with her tuneful companion, and some women even cried with pleasure to hear it, and many said, "It makes me quite forget my pain." This is an example of the pleasure caused by "little things." If any of our readers follow my suggestion, and witness the pleasure they give, I know they will thank me in their hearts for a humble word in due season.

[This idea is excellent. We shall be glad to receive offers of musical boxes. Musical boxes for distribution, or the money-value of a box, say from £5 5s. to £12 12s., from those of our readers who have money but no instruments.—Ed.]

### A NEURASTHENIC HOME.

"Cambridge" writes: What do you think would be the prospects of a home for neurasthenic cases in the vicinity of London? I propose to take a good-sized house, with garden, and receive girls suffering from different forms of hysteria. It would be under the direction of a medical man, the chief idea being to institute cheerful and useful occupations as daily duties in addition to such remedies as Swedish exercises, &c. The doctor's prescription to assign the daily rule of life to each patient. There would be classes and lectures in connection with the treatment, and the usual period of residence would be three months.

### Examination Questions.

The prize for November is awarded to Miss Ditmas (Sister Faith), of Eastbourne, to whom we have sent Shaw's "Antiseptics in Obstetric Nursing." The following deserve honourable mention: Nurse Clark (of Edinburgh), Nurse Edith Gibson, Sister K. Drew, Miss Chambers, Nurse Esther Payne, Nurse Alice Grad, Nurse Maggie Stocks, Nurse Josephine Taylor, and Nurse Becks. All these cases are so interesting that we hope to give a brief resumé of them later on; next week we hope to give Nurse Clark's notes in full; unfortunately, it is impossible to reproduce Sister Faith's extremely neat and complete method of case-taking. As this is a frivolous time, we offer a book to the first person who sends us correct solutions of the following riddles:—(1) What drug is remarkable for lengthening the life of scholars? (2) What is the difference between cantharides and a lottery ticket? (3) What two opposite diseases are caused by match-making? (4) Of what bird does paracentesis remind you? (5) Why does orthopnœa cause honesty? (6) Why is pyrosis like a love affair?

### Notes and Queries.

### Query.

(17) *Nursing in Prisons.*—Are there proper wards in prison where the prisoners, when sick, have trained attendance? I should like to devote myself to work in a prison ward.—*Nurse W.*

### Answers.

*Brisbane and Others.*—Next week, no room this.

*Maggie.*—The London Hospital, Whitechapel, two years; the Southern Hospital, Liverpool, one year; Queen's Hospital, Birmingham, two years; Dewsbury Infirmary, two years.

*Local High Temperature.*—Please send us further notes of your interesting case.

*Conjuring.*—Entertainers of all sorts can be had from the Civil Service Stores at a reasonable rate.—*Matron.*

*Guild of St. Veronica.*—The address of the secretary of this nursing guild is Miss F. Robertson Macdonald, 9, Great Bedford Street, Bath. A stamped envelope should be sent by those who desire an answer.

*Christmas Competitions.*—Parcels received from Miss Clarke, Nurse Chandler, Nurse Elms, Miss E. M. Jones, Nurse Dudley, and Nurse Saunders.

*Stella.*—"The Nurse's Dictionary," published at this office, price 2s.; or Keating's "Medical Lexicon," published by Lewis, price 3s. 6d.

*F. Kennedy.*—Bristol Hospital for Children, certificate at the end of two years; Gateshead-on-Tyne Hospital for Children, one year; Pendlebury Hospital, near Manchester, one year. We know nothing of the Liverpool Children's Hospitals.

### The Nurses' Bookshelf.

### A NURSES' DICTIONARY.*

Judging from the enquiries we have received and are continually receiving, this book should be popular. It supplies a space in the bookshelf of the household which has been too long vacant. We have made a careful examination of its pages, and can cordially recommend it as containing accurate information pithily put in simple language which everybody can understand. It is superior to most of the dictionaries of medical terms in the fact that it not only contains definitions of the principal medical and nursing terms and abbreviations, but a description of instruments and drugs and their use. Here will be found an account of the various foods; an explanation of many accidents and forms of treatment and operation, so that a nurse will now be able, without difficulty, to under stand intelligently what she hears and sees. Thus under the word Enema is a description of the instrument in its various forms, an account of how to use it, the precautions to be taken, and the constituent parts of the various enemata most commonly used. Epilepsy is defined as a disease, the points which the nurse ought to note before and after the seizure, the condition of the patient, and the precautions to be taken to prevent the patient from injuring himself are all dealt with. The definition of rheumatism is followed by directions as to the nurse's duties in regard to a patient so afflicted; the use of poultices is followed by a description of the various kinds in common use and the best methods of preparing them. The Dictionary contains many practical hints, and as a specimen we cannot do better than quote the following: "Convalescence—The period of returning strength after an illness. The nurse needs to amuse her patient, prevent rash deeds, or fatigue arising from too many visitors; supply light, nutritive food at frequent intervals; avoid all talk about the past illness, and watch for a relapse." There are several similar spoonfuls of sound sense. The dictionary is wonderfully free from errors, but we must correct one to prevent misapprehension. Under Fahrenheit, the normal heat of the human body is given as 94 deg., instead of 98·4 deg. This error is probably caused by the printer having dropped the figure 8, but nurses should note and correct it in their copies. Every mother, as well as every nurse, should provide herself with a copy of "The Nurses' Dictionary," because both in practice will find it indispensable. The dictionary is excellently printed, and we congratulate the compiler on a good piece of work, which badly wanted doing.

* "The Nurses' Dictionary of Medical Terms and Nursing Treatment, compiled for the use of nurses, and containing descriptions of the principal Medical and Nursing Terms and Abbreviations, Instruments, Drugs, Diseases, Accidents, Treatment, Physiological Names, Operations, Foods, Appliances, &c., encountered in the Ward or Sick Room." By Honnor Morten. (London: The Hospital (Limited), 140, Strand, W.C.) Price 2s.

### The Nurses' Bed.

We have received 5s. from "Wigtonshire," together with a kind letter for which we must return special thanks. Also 6s. collected by a male attendant, which raises a dreadful thought in our mind that we had in this matter quite forgotten our nursing brothers, and where they are to go if they are in need of rest and change. We fear that often male attendants do not receive all the sympathy and help they might from their sisters in arms. And Miss Holditch requests us to acknowledge £1 5s. collected by Nurse Taylor, of Maidstone; and Miss E. Clarke sends 1s. 6d. towards the furnishing.

### "Mike."

"We need Love's tender lessons taught
As only weakness can;
God hath His small interpreters,
The child must teach the man."—*Whittier.*

It was growing dusk, the short winter's afternoon was nearly over, and the weary sufferers in the long hospital ward were longing for tea-time, when the big door opened suddenly, and the young house-surgeon walked briskly up the room. But there was a tired strained look on his white face, as he went his round, that showed he was worried. Only that afternoon he had been a spectator at the marriage of the woman he loved, and he was thinking bitterly that life was not worth living now, and idly wishing himself dead. He was about to leave the ward, when the nurse reminded him of the dying boy in the side-room, and with a tired sigh he followed her into the little room, just in time to overhear the boy moan, "O God! let me die, if you have nothing that you want me to stop for." He was a boy of about twelve, a pale, half-starved looking child with large hungry eyes, and a look of patient suffering on a weary little face, that told its own tale of poverty and neglect. But when the doctor bent over him, the face lit up with joy, and he said faintly, "Well, doctor." "My dear little fellow, God is going to answer your prayer," said the doctor, with a tender smile into the pathetic little face. But he added suddenly, "If God wanted you to live and bear your pain for a while longer, would you be willing?" A wistful look crept into the boy's eyes, but he answered trustfully, "Yes, sir; He knows best." And with something like a sob in his voice, the doctor whispered, "O God! give me this child's faith"—and the prayer was answered.

### Where to Go.

The next lecture at the Nurses' Club will be given by Dr. Tom Robinson, on Friday evening, December 18th, at a quarter to eight. Subject, "Parasitism." A few tickets to non-members 6d. each.

Free organ recitals are given every Sunday afternoon at the Albert Hall.

Mr. Corney Grain is giving daily, at St. George's Hall, an amusing sketch called "The Diary of a Tramp"; it is well worth hearing.

### Appointment.

Rotherham Infirmary.—Miss Sarah Stevenson has been appointed Matron of the infirmary, Rotherham. Miss Stevenson was trained at Glasgow Royal Infirmary. She went from thence to the Children's Hospital, Glasgow. Miss Stevenson had charge first of the children's wards, and afterwards of the ophthalmic wards in the Bristol Royal Infirmary. She joined St. George's Home, Sheffield, two years ago, and has done admirable work there.

### Amusements and Relaxation.

SPECIAL NOTICE TO CORRESPONDENTS.

**Fourth Quarterly Word Competition commenced October 3rd, ends December 26th, 1891.**

The word for dissection for this, the ELEVENTH week of the quarter, being

"YULE-TIDE."

| Names. | Dec. 3rd. | Totals. | Names. | Dec. 3rd. | Totals. |
|---|---|---|---|---|---|
| Lightowlers | 10 | 425 | Jenny Wren | 12 | 339 |
| Bonne | 11 | 414 | Darlington | 10 | 420 |
| Morico | 11 | 486 | Nurse G. P. | — | 99 |
| Robes | — | 143 | Hetty | 11 | 338 |
| Dulcamara | 11 | 433 | Janet | — | — |
| Psyche | — | 7 | Jackanapes | 13 | 339 |
| Agamemnon | 13 | 458 | Ex Nurse | — | 99 |
| Nurse J. S. | 10 | 407 | | | |

All letters referring to this page which do not arrive at 140, Strand, London, W.C. by the *first post on Thursdays*, and are not addressed PRIZE EDITOR, will in future be disqualified and disregarded.

## Figures and Tables

**Figure f1:**